# Evaluation of the transmission-blocking potential of *Plasmodium vivax* antigen Pvg37 using transgenic rodent parasites and clinical isolates

**DOI:** 10.3389/fcimb.2025.1529770

**Published:** 2025-01-24

**Authors:** Di Zhang, Yan Zhao, Dongyan Liu, Fei Liu, Pengbo Liu, Biying Zhang, Zifang Wu, Wanlapa Roobsoong, Sirasate Bantuchai, Sataporn Thongpoon, Piyarat Sripoorote, Meilian Wang, Liwang Cui, Yaming Cao

**Affiliations:** ^1^ Department of Immunology, College of Basic Medical Sciences, China Medical University, Shenyang, China; ^2^ ShengJing Hospital of China Medical University, Department of Gastroenterology and Medical Research Center, Shenyang, Liaoning, China; ^3^ Mahidol Vivax Research Unit, Faculty of Tropical Medicine, Mahidol University, Bangkok, Thailand; ^4^ Department of Pathogen Biology, College of Basic Medical Sciences, China Medical University, Shenyang, China; ^5^ Department of Internal Medicine, Morsani College of Medicine, University of South Florida, Tampa, FL, United States

**Keywords:** *Plasmodium vivax*, transmission-blocking vaccine, polypeptide, transgenic parasite, gametocyte

## Abstract

**Background:**

*Plasmodium vivax* is a major cause of malaria, particularly outside Africa, necessitating effective strategies for public health management. Transmission-blocking vaccines (TBVs) have shown the potential to inhibit malaria transmission by targeting antigens expressed in sexual-stage parasites. Pbg37, a conserved protein expressed in sexual stages from gametocyte to ookinete in the rodent parasite *P. berghei*, is a viable target for TBV development.

**Methods and findings:**

In this study, we constructed a transgenic strain, *TrPvg37Pb*, expressing Pvg37 using the *P. berghei ΔPbg37* strain. Initial findings demonstrated that the replacement of *Pbg37* with the exogenous *Pvg37* did not impact parasite growth or development. Notably, Pvg37 was expressed during the gametocyte to ookinete development and was associated with the plasmic membrane, similar to Pbg37. To evaluate the potential of Pvg37 as a TBV candidate, we synthesized two Pvg37 polypeptides and immunized rabbits to generate antibodies. *In vitro* experiments demonstrated that anti-Pvg37-P2 antibodies significantly inhibited the formation of male gametes and ookinetes in the transgenic *TrPvg37Pb* parasite. Additionally, in mosquito feeding assays, mosquitos feeding on *TrPvg37Pb*-infected mice passively transferred with anti-Pvg37-P2 antibodies showed a significant 80.2% decrease in oocyst density compared to the control group. Furthermore, in direct membrane feeding experiments using four clinical *P. vivax* isolates, the anti-Pvg37 antibodies significantly reduced oocyst density by 28.6–50.4%.

**Conclusion:**

Pvg37 is a promising candidate for *P. vivax* TBV development, deserving further research and optimization to enhance its immunogenicity and transmission-blocking activity.

## Introduction

Malaria is a severe parasitic disease caused by *Plasmodium* parasites. According to the World Health Organization’s World Malaria Report 2023, there were 249 million cases worldwide in 2022, an increase of 5 million cases compared with 2021 (WHO, 2023). *Plasmodium vivax* is a major cause of malaria outside of Africa and accounts for about 72% of all cases in Southeast Asia and the Americas (Flannery et al., 2019). Managing and treating *P. vivax* malaria is more challenging than *P. falciparum* malaria, as it produces dormant hypnozoites in the liver that are responsible for relapse (Adams and Mueller, 2017; Flannery et al., 2022). Currently, primaquine and tafenoquine are utilized to clear hypnozoites, but they are contraindicated in individuals with glucose-6-phosphate dehydrogenase (G6PD) deficiency due to the risk of hemolysis (Lacerda et al., 2019; Llanos-Cuentas et al., 2019; Thriemer et al., 2021). The Malaria Eradication Research Agenda (MalERA) considers that interrupting malaria transmission is a key measure for malaria elimination, with transmission-blocking vaccines (TBVs) uniquely suited for this task.

In membrane-feeding assays, antibodies targeting antigens consumed in a blood meal can suppress the growth of parasites within mosquito vectors. TBV candidate antigens are primarily expressed on the surface of the mosquito-stage malaria parasite (Miura et al., 2019). Thus, they are less susceptible to selective pressure from the vertebrate immune system and display lower levels of polymorphism. According to their expression patterns, TBV antigens fall into two categories. Pre-fertilization antigens such as P48/45 and P230 are expressed in gametocytes and gametes, while post-fertilization antigens such as P25 and P28 are expressed on the surfaces of zygotes and the maturing ookinetes (Hisaeda et al., 2001; van Dijk et al., 2001; Doi et al., 2011; Arévalo-Herrera et al., 2015). Although TBV research has received considerable attention, only a limited number of candidate antigens have been identified (de Jong et al., 2020), especially for *P. vivax*. These include pre-fertilization antigens Pvs230, Pvs48/45, and PvHAP2, post-fertilization antigens Pvs25 and Pvs28, and the mosquito midgut antigen AgAPN1 (Malkin et al., 2005; Tachibana et al., 2012, 2015; Tentokam et al., 2019). Recombinant Pvs25H expressed in *Saccharomyces cerevisiae* has been evaluated in Phase I clinical trials with alum or Montanide ISA51 as an adjuvant (Malkin et al., 2005; Wu et al., 2008). It has been shown that >25% of endemic populations showed natural antibody responses to the Pvs230 domain 1 (Tentokam et al., 2019). In *P. falciparum*, Pfs230 regarded as a homologue of Pvs230 is considered a more promising TBV candidate (Healy et al., 2021). A vaccine targeting the first domain of Pfs230 has demonstrated a stronger TBA than the comparable Pfs25 vaccine and is currently in Phase II field trials in Mali (Duffy, 2022). Another pre-fertilization antigen, PvHAP2, showed transmission-reducing activity (TRA) of 40.3–89.7% in a direct membrane feeding assay (DMFA) (Qiu et al., 2020). Therefore, there is a clear priority in TBV antigen discovery for *P. vivax*.

We have identified Pbg37 as a conserved sexual-stage antigen across the genus *Plasmodium*. It was expressed intracellularly in gametocytes, but the protein became membrane-associated during gametogenesis and zygote-ookinete development (Liu et al., 2018). Pbg37 is essential for sexual development, as its deletion led to a significant reduction in gametocytemia and oocyst numbers in mosquitoes. Direct feeding of mosquitoes on mice immunized with recombinant Pbg37 resulted in a 49.1% reduction in oocyst density. This TRA and the conservation of Pbg37 in *Plasmodium* prompted us to investigate the TB potential of its ortholog in *P. vivax*, Pvg37. By replacing *Pbg37* with *Pvg37*, we generated a transgenic *P. berghei* parasite line expressing Pvg37. Using this transgenic parasite and clinical *P. vivax* isolates, we conducted mosquito-feeding assays and demonstrated that antibodies against Pvg37 also possessed substantial TRA.

## Materials and methods

### Mice, parasites and mosquitoes

Female BALB/c mice and New Zealand white rabbits were purchased from the Beijing Animal Institute. The *P. berghei* ANKA strain 2.34 was maintained by serial passage and used for challenge infection as described previously (Bai et al., 2023). The *Δpbg37* parasite used for generating a transgenic parasite expressing Pvg37 was from an earlier study (Liu et al., 2018). Adult (3-5 days old) *Anopheles stephensi* and *An. dirus* mosquitoes were fed on a 10% (w/v) glucose solution and kept in an insectary under 25°C ± 1°C and 50 – 80% relative humidity, with a 12 h light and dark cycle. All animal procedures were carried out per the welfare and ethical review standards of China Medical University.

### Construction of a *P. berghei* strain expressing pvg37

The *TrPv37Pb* line was generated by inserting the complete *Pvg37* open reading frame (ORF, 1053 bp) tagged with 3×HA at its C-terminus into the pL0034 vector at the *ApaI* and *XhoI* sites. The *Pvg37* ORF was flanked by the 5’ UTR and 3’ UTR of the *Pbg37* gene. Ten micrograms of the plasmid were digested with *ApaI* and *XhoI* and then electroporated into purified *Δpbg37* schizonts using a Nucleofector system. Subsequently, the parasites were injected intraperitoneally into two mice. After 24 h, the *TrPv37Pb* line was selected through gavage in mice using 5-fluorocytosine (20 mg/mL in water). To confirm the proper integration of the *Pvg37* gene at the *Pbg37* locus in the *P. berghei* genome, PCR analysis was performed using specific primers (**Supplementary Table S1**). The *TrPv37Pb* parasites were then cloned using a limiting dilution technique.

### Phenotypic analysis of the *TrPvg37Pb* parasites

To investigate the impact of *Pvg37* on parasite development, we compared the development among the (wild-type) WT *P. berghei*, *TrPvg37Pb*, and *Δpbg37* lines. Three groups of BALB/c mice (3 mice per group) were intraperitoneally injected with 1×10^6^ infected red blood cells (iRBCs) of the respective parasite clones. Asexual parasitemia levels were monitored on days 3, 5, 7, 9, and 11 post-infections by examining Giemsa-stained thin blood smears. The number of mature gametocytes per 100 parasites was determined during the parasitemia range of 10–20%. In each mouse, 100 mature gametocytes were differentiated into male and female gametocytes based on morphological characteristics to establish the gametocyte sex ratio. Following induction for gametogenesis at 25°C for 15 min, the culture was transferred onto a coverslip, and exflagellation centers were counted under a phase-contrast microscope at 400× magnification. To observe ookinete formation, 10 μL of infected blood containing equal gametocyte counts were mixed with the ookinete culture medium in a total volume of 50 μL and maintained at 19°C for 24 h. The gametocyte counts were normalized according to gametocytemia. The ookinete number in 0.5 μL of culture was counted using an IFA, with ookinetes stained with a monoclonal antibody (mAb) against Pbs21.

### Pvg37 polypeptide synthesis and polyclonal antibody generation

The Pvg37 protein fragments spanning amino acids 25 to 38 and 55 to 68 were synthesized as polypeptides (Genescipt, China), namely Pvg37-P1 and Pvg37-P2, respectively, which were conjugated to keyhole limpet hemocyanin (KLH) for immunization. Three rabbits were subcutaneously immunized with 500 μg of Pvg37 peptides emulsified in Freund’s complete adjuvant. Three booster immunizations were performed at weeks 2, 5, and 8 after emulsification with 250 μg of Pvg37 peptides and incomplete Freund’s adjuvant. The immune serum was collected 10 days after the last immunization. IgGs were purified from Pvg37 immune and pre-immnue sera, respectively, using Protein A columns. The concentrations of anti-Pvg37 and pre-immune control antibodies were determined using the BCA Protein Assay kit.

### Enzyme-linked immunosorbent assay

ELISA was utilized to determine antibody titers of sera. A 96-well plate was coated with polypeptides Pvg37-P1 and Pvg37-P2 in 0.05 M sodium carbonate buffer (pH 9.6) and incubated overnight at 4°C. The samples were then washed three times with PBST (0.05% Tween-20, 0.1 M PBS) and incubated with 1% BSA (Sigma) for 1 h at 37°C. Following another round of washing with PBST, the anti-Pvg37 peptide sera and negative control sera were diluted in PBS containing 1% BSA at multiple proportions ranging from 1:1000 to 1:512000. The samples were incubated at 37°C for 2 h and washed three times with PBST. HRP-labeled sheep anti-rabbit IgG, diluted in 3% BSA (1:5000), was added to the 96-well plates, and the samples were incubated at 37°C for 1 h. Subsequently, the plates were washed five times, and tetramethyl-benzidine was added for color development in the dark for 10 min. The reaction was stopped by adding 2 mM H_2_SO_4_, and the absorbance value at 490 nm was measured. The final dilution value was considered to be higher than the mean + 3 × standard deviation (cut-off value) of the pre-immune control sera.

### Indirect immunofluorescence assay

IFA was performed on gametocytes, gametes, zygotes, retorts, and ookinetes. TrPvg37Pb parasites were fixed with 4% paraformaldehyde and 0.0075% glutaraldehyde in PBS for 30 min at room temperature. Then, the parasites were washed twice with PBS. After being permeabilized with 0.1% Triton X-100, parasites were blocked with PBS containing 3% BSA for 1 h at 37°C. The rabbit anti-Pvg37-P2 sera (1:200) in PBS containing 3% BSA were added into parasites for 1 h at 37°C. All parasites were co-incubated with mouse antisera against PbMSP1 (1:500), Pbs47 (1:500), Pbα-tubulin (1:500), and Pbs21 (1:500) as specific markers for schizonts, female gametocytes/gametes, male gametocytes/gametes, and zygotes/ookinetes, respectively. These antisera were self-made and have been previously reported (Zheng et al., 2024). After washing the slides with PBS, Alexa Fluor 488-conjugated anti-rabbit IgG secondary antibodies (1: 500, Invitrogen) and Alexa-555 conjugated goat anti-mouse IgG secondary antibodies (1: 500, Abcam) were added into the parasites for 1 h at 37°C. WT ookinetes were used as the negative control. Images were acquired using a Leica SP8 confocal laser scanning microscope. For comparison, mouse anti-HA mAb (1:500, abclone) was also used to probe parasites to determine the expression stage of Pvg37. Furthermore, the localization of Pvg37 protein on the *P. vivax* gametocytes was confirmed using anti-Pvg37-P2 IgGs and isolating gametocytes from clinical samples of *P. vivax.*

### Purification of *TrPvg37Pb* parasites at different stages

Different gradient Nycodenz was used to isolate and purify TrPvg37Pb parasites at various stages. When the parasitemia reached 3-5%, mouse blood was collected and mixed with schizont culture medium (RPMI 1640, 50 mg/L penicillin, 50 mg/L streptomycin, 100 mg/L neomycin, 25% fetal bovine serum, and 6 U/mL heparin). The mixture was cultured at 37°C for 20 h. Schizonts were subsequently isolated and purified on a 56% (v/v) Nycodenz gradient. For purifying sexual stages, mice were treated with sulfadiazine (Sigma, Burlington, USA, 20 mg/L) for 2 days to eliminate asexual blood stages when the parasitemia ranged between 10 and 20%. When parasites reached the gametocyte stage, blood was collected and mixed with PBS at 4°C to prevent gametocyte activation. Gametocytes were then isolated and purified on a 48% (v/v) Nycodenz gradient. To obtain ookinetes, 1 mL of blood was mixed with 9 mL of ookinete medium (50 mg/L penicillin, 50 mg/L streptomycin, 100 mg/L neomycin, 20% fetal bovine serum, 1 mg/L heparin, pH 8.3) and cultured at 19°C for 24 h. Ookinetes were then isolated and purified using a 62% (v/v) Nycodenz gradient. Finally, purified parasites from each stage mentioned above were washed twice with PBS.

### Western blot analysis

To determine the expression of Pvg37 at different stages, purified schizonts, gametocytes, and ookinetes were treated with 0.2% saponin to lyse erythrocytes. After three washings with PBS, parasites were treated with RIPA lysis buffer containing phenylmethylsulfonyl fluoride three times to extract total proteins. Protein concentrations were determined using the BCA Protein Assay kit. Equal parasite proteins (20 μg/lane) were separated by 10% SDS-PAGE and transferred to a 0.22 μm PVDF membrane. Then, the PVDF membrane was blocked with TBST containing 5% skim milk for 1 h at 37°C. After blocking, the PVDF membrane was probed with anti-Pvg37 rabbit immune sera (1:200) and anti-rHSP70 sera (1: 1000), and after three washing with TBST, HRP-conjugated goat anti-rabbit IgG antibodies (1: 10000, Invitrogen) as the secondary antibodies were added. The blots were then detected by an ECL Western Blotting Kit (Beyotime).

### Quantification of TB activity

The TB potential of Pvg37 was evaluated using two assays: an *in vitro* ookinete formation assay and a direct mosquito feeding assay. The *in vitro* assay employed various dilutions of the immune sera. Groups of BALB/c mice (three mice per group) were injected intraperitoneally with 1×10^6^ iRBCs of the *TrPv37Pb* line. The exflagellation of male gametocytes was quantified using the method mentioned above. Purified pre-immune IgGs or anti-Pvg37-P2 IgGs were added into the ookinete culture medium at a final concentration of 0.2, 1.0, and 2.0 µg/µL and mixed with 10 μL of blood from mice infected with the *TrPvg37Pb* parasites, resulting in a total volume of 50 μL. The number of exflagellation centers was observed in each microscope field at 400x magnification. Ookinete cultures were incubated at 19°C for 24 h, and mature ookinetes in 0.5 μL of culture were counted using a fluorescence microscope (100× oil objective). For antibody transfer experiments, 150 µL of purified anti-Pvg37-P2 IgGs were injected into the tail veins of the mice one hour prior to *An. stephensi* mosquito direct feeding. Twelve days after feeding, mosquitoes were dissected to assess the number of oocysts using a compound microscope at 200× magnification.

### Direct membrane feeding assay

We conducted DMFA using blood samples from volunteers infected with *P. vivax*. Prior to participation, informed written consent was obtained from four volunteers. Parasitemia was estimated using Giemsa-stained films. The anti-Pvg37-P2 antibody and the negative control antibody were diluted 1:1 with 90 μl of heat-inactivated (complement negative) healthy human AB+ serum, resulting in a total volume of 180 μl. The diluted antibodies were then mixed with RBCs collected from *P. vivax* malaria patients in a 1:1 ratio. Pooled blood samples were incubated at 37°C for 15 min and then introduced into a membrane feeder. Approximately 100 *An. dirus* mosquitoes (starved for 12 h before the experiment) were fed with the blood samples for 30 min using the membrane feeder maintained at 37°C. Unfed mosquitoes were subsequently removed, and fed mosquitoes were allowed to feed on cotton pads dipped in a 10% sucrose solution at 27°C and 80% relative humidity for one week. For each group, 20 mosquitoes were dissected to count oocysts.

### Analysis of genetic polymorphisms

Genomic DNA from the four *P. vivax* isolates used in the DMFA was extracted using a QIAamp DNA Blood Mini kit (Qiagen, Germany). The *pvg37* DNA fragment encoding aa 1–182 was amplified by PCR with primers designed based on the Sal-I sequence (**Supplementary Table S1**). The purified PCR products were sequenced using the ABI Prism BigDye™ cycle sequencing kit (Applied Biosystems, Thermo Fisher Scientific). The sequences were aligned using ClustalW in MEGA7.0.26.

### Statistical analyses

Statistical analyses were conducted using SPSS software version X (SPSS Inc., USA). Ordinary one-way ANOVA was used to compare the groups in terms of asexual parasitemia, gametocytes, sex ratio, exflagellation, and ookinete numbers. Mann-Whitney U test was employed to analyze oocyst density (oocyst number per midgut), while Fisher’s exact test was used to analyze infection prevalence. The results are presented as the mean ± SD. A significance level of 0.05 was considered statistically significant.

## Results

### Generation of transgenic *P. berghei* parasite *TrPvg37Pb*

**Figure 1:**
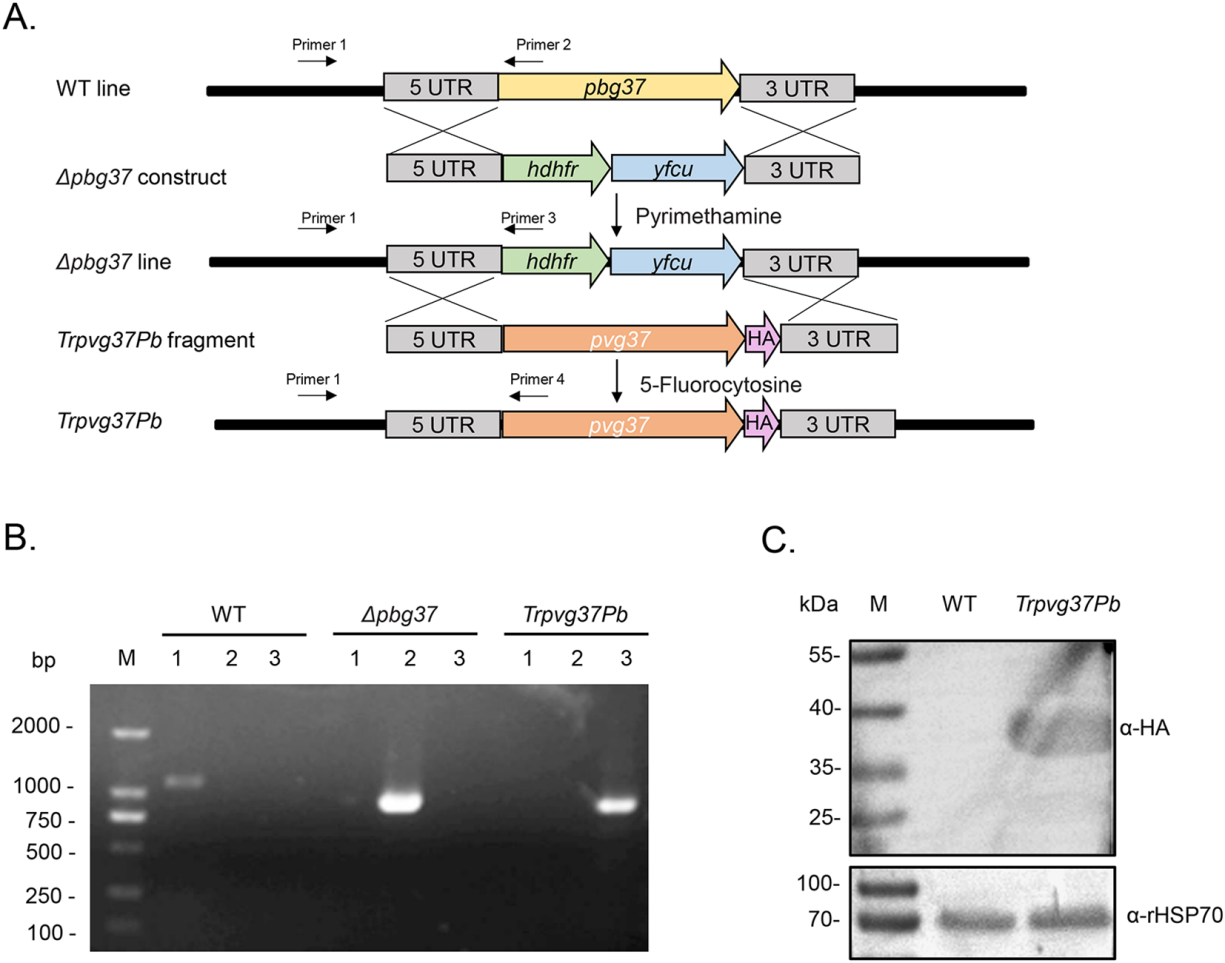
Generation of the *TrPvg37Pb* parasites. **(A)** Schematic diagram illustrating the construction of the *TrPvg37Pb* transgenic parasite through double homologous recombination, wherein the *hdhfr::yfcu* selection cassette in *Δpbg37* parasites is replaced with the *pvg37* gene. Primers 1 – 4 used for diagnostic PCR are depicted. **(B)** PCR identification of the *TrPvg37Pb* transgenic line. Primers 1 + 2 were employed to confirm wild-type (WT) locus, primers 1 + 3 were used to identify *pbg37* deletion, while primers 1 + 4 were utilized to confirm successful replacement of *pvg37* gene in *P. berghei* line. The resulting PCR products showed distinct band sizes: lanes 1, PCR with primers 1 + 2 (1104 bp); lanes 2, PCR with primers 1 + 3 (868 bp); lanes 3, PCR with primers 1 + 4 (796 bp). **(C)** Western blot analysis for identifying WT and *TrPvg37Pb* parasites using anti-HA monoclonal antibody (top), while HSP70 was employed as a protein loading control (bottom).

The TrPvg37Pb transgenic strain was constructed using the Gene Insertion and Marker Out (GIMO) technique. This approach involves replacing the drug resistance gene in the obtained *Δpbg37* strain with the *pvg37* gene, which is tagged with a 3×HA tag, under negative selection with 5-fluorocytosine (**Figure 1A**). The genotypes of WT, *Δpbg37*, and *TrPvg37Pb* were identified by PCR amplification using primers 1 and 2, 1 and 3, and 1 and 4, respectively (**Figure 1A**). Diagnostic PCR results confirmed successful recombination of the *pvg37* gene as specific bands were only amplified from corresponding parasite gDNA samples (**Figure 1B**). Additionally, Pvg37-HA protein expression in transgenic *P. berghei* parasite lines was confirmed by Western blotting using the anti-HA mAb. A protein band of approximately 39 kDa was detected in the *TrPvg37Pb* parasites but not found in WT parasites (**Figure 1C**).

### Pvg37 expression restores the normal development of *Δpbg37* parasites

**Figure 2:**
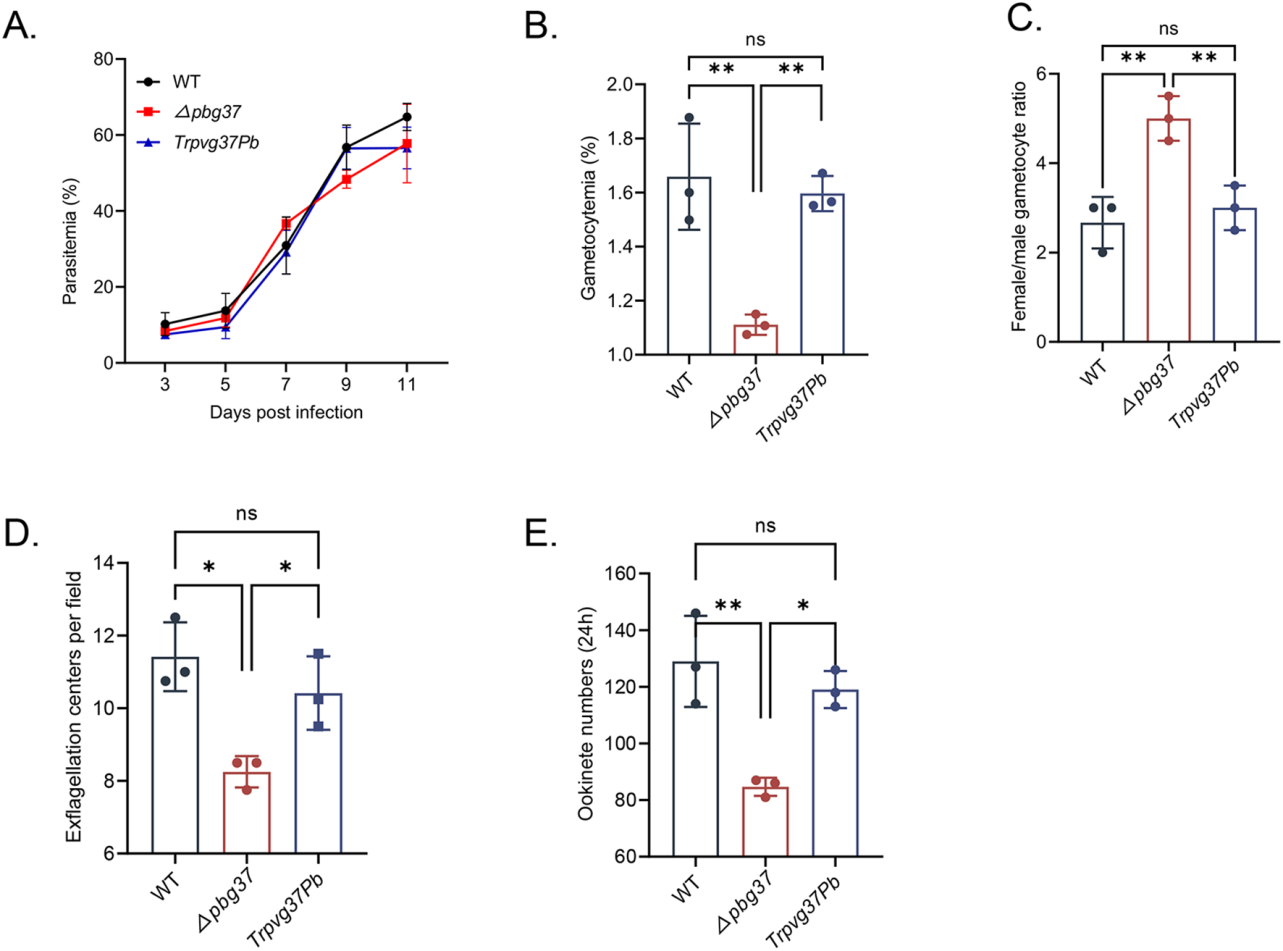
Phenotypic analysis of *TrPvg37Pb* parasites. **(A)** Parasitemia of mice infected with WT, *Δpbg37*, or *TrPvg37Pb* parasites. **(B)** Gametocytemia (percentage of gametocyte in 100 RBC). **(C)** Female/male gametocyte ratios. **(D)** Number of exflagellation centers per field at 400× magnification. **(E)** Quantification of ookinete numbers in 0.5 μl of *in vitro* culture through immunostaining with anti-Pbs21 mAb. All experiments were performed in triplicate. Error bars indicate mean ± SD. *P < 0.05, **P < 0.01 (one way ANOVA). "ns" denotes no significance.

The impact of complementing Pvg37 on the growth and development of *P. beighei* was assessed through phenotype analysis performed on *TrPvg37Pb*, *Δpbg37*, and WT strains. Equal numbers of parasite-infected RBCs for each strain were injected into BALB/c mice via the tail vein. The results showed no significant difference in asexual-stage parasitemia among *TrPvg37Pb, Δpbg37*, and WT parasites (**Figure 2A**). However, the *Δpbg37* strain exhibited significantly lower gametocytemia and a higher female/male gametocyte ratio (**Figures 2B, C**). Furthermore, compared to WT parasites, the *Δpbg37* strain displayed a significant reduction of 27.8% in exflagellation centers and 34.4% in ookinete formation (**Figures 2D, E**), consistent with previously published data (Liu et al., 2018). Notably, no significant differences were observed between the TrPvg37Pb strain and WT strain in terms of gametocyte formation, exflagellation centers, or ookinete production (**Figures 2B-E**). These findings indicate that substituting Pbg37 with Pvg37 does not affect the development of both asexual and sexual stages of *P. berghei*. Moreover, Pvg37 compensates for the abnormal phenotype caused by *pbg37* deletion, suggesting a functional equivalence between Pvg37 and Pbg37.

### Production of high titers of antibodies against Pvg37

**Figure 3:**
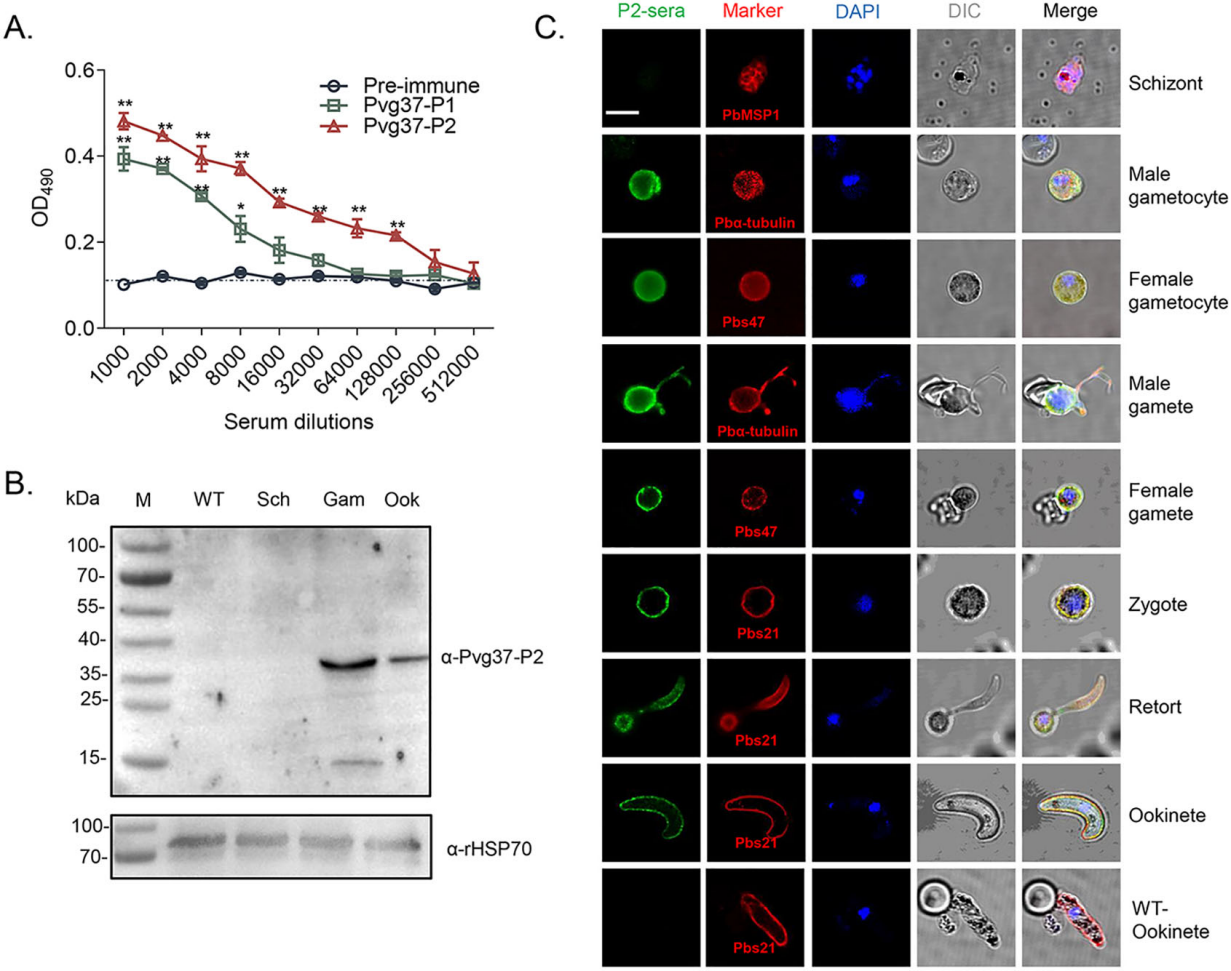
Pvg37 expression and localization in *TrPvg37Pb* parasites. **(A)** The total antibody titer of anti-Pvg37-P1 and anti-Pvg37-P2 sera at 10 days after the final immunization was analyzed by ELISA. Mean of control pre-immune sera + 3 × SD is shown by the broken lines. IgG titers were determined as the highest dilution of anti-Pvg37 sera where OD_490_ values were above the cut-off values. Cut-off value was defined as that of the pooled sera from control mice. The error bar shows mean ± SD. *P < 0.05, **P < 0.01 (Student’s t-test). **(B)** Western blot analysis was performed on lysates containing 20 μg of protein per lane derived from the control WT parasites, as well as purified schizonts (Sch), gametocytes (Gam), and ookinetes (Ook) of the *TrPvg37Pb* parasites. The proteins were probed with anti-Pvg37-P2 IgGs (top), while HSP70 served as a loading control for protein quantification (bottom). **(C)** Immunofluorescence assays were conducted on *TrPvg37Pb* parasites at various stages using anti-Pvg37-P2 IgGs. The WT ookinete was used as control. The scale bar represents 5µm.

The Pvg37 protein possesses seven transmembrane domains similar to Pbg37, and multiple sequence alignment revealed a high degree of conservation among *Plasmodium* species. Two highly conserved regions in Pvg37 were identified at amino acids 25-38 and 55-68, which showed abundant B-cell antigenic epitopes (Liu et al., 2018). Therefore, we opted to utilize these two highly conserved regions, namely Pvg37-P1 and Pvg37-P2, for peptide synthesis. Polyclonal antibodies against Pvg37-P1 and Pvg37-P2 were generated by immunizing rabbits separately. ELISA showed that the final antibody titers for Pvg37-P1 and Pvg37-P2 reached 1:8000 and 1: 128000, respectively (**Figure 3A**). Remarkably, the anti-Pvg37-P2 sera exhibited significantly higher antibody titers than the anti-Pvg37-P1 sera. Thus, we selected the Pvg37-P2 antibodies for subsequent experiments. IgG concentrations purified from the Pvg37-P2 immunized serum and pre-immunized serum were 11.667 and 14.168 μg/μL, respectively, which were adjusted to 10 mg/mL with PBS for transmission-blocking assessment.

### Pvg37 is expressed and localized in sexual stages

To confirm the expression and localization of the Pvg37 protein in transgenic parasites, we purified TrPvg37Pb parasites at different stages, including schizonts, gametocytes, gametes, and ookinetes. Western blot using the rabbit anti-Pvg37-P2 sera detected a ~37 kDa protein band in *TrPvg37Pb* gametocytes and ookinetes but not schizonts. The expression level in gametocytes appeared higher than in ookinetes (**Figure 3B**).

We examined the subcellular localization of Pvg37 protein in *TrPvg37Pb* parasites by IFA with rabbit anti-Pvg37-P2 IgGs. Fluorescent signals were observed in the cytosol and at the plasma membrane of gametocytes (**Figure 3C**). During the gametogenesis of microgametocytes, signals were prominently observed along the flagellas. In subsequent development, Pvg37 was specifically associated with the plasma membranes of gametes, zygotes, retorts, and ookinetes (**Figure 3C**). No signal indicating Pvg37 expression was detected in *TrPvg37Pb* schizonts or WT ookinetes. Since the Pvg37 protein was C-terminally tagged with a 3×HA tag in the *TrPvg37Pb* parasite, we also performed IFA with a mouse anti-HA mAb and obtained similar results as with anti-Pvg37-P2 IgGs (**Supplementary Figure S1**). Overall, these findings demonstrate that Pvg37 exhibited a plasma membrane localization pattern during gamete–ookinete transition, similar to Pbg37.

### Anti-Pvg37 IgGs show transmission reduction activity in *TrPvg37Pb* parasites

**Figure 4:**
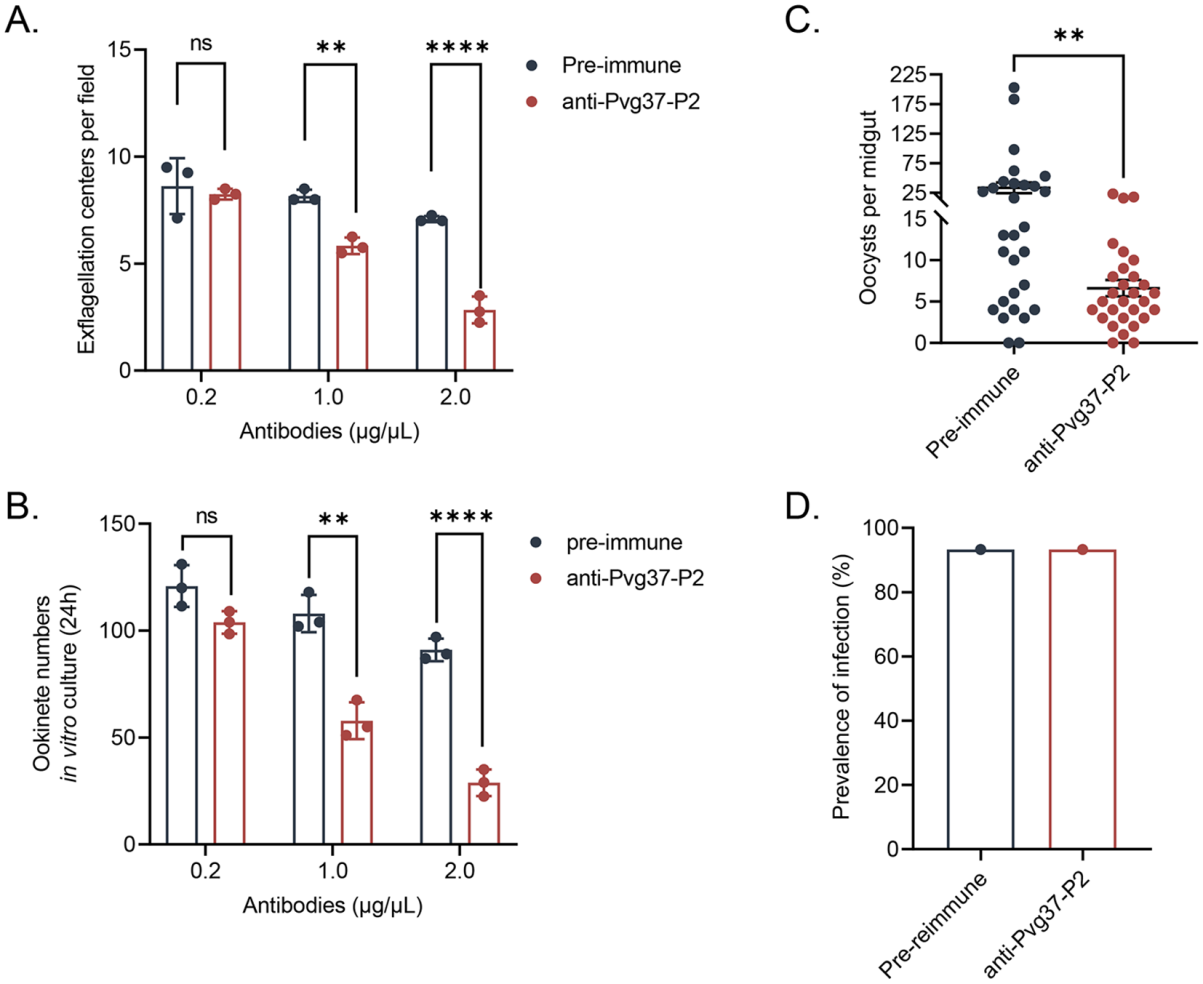
Evaluation of the transmission blocking effect of anti-Pvg37-P2 IgGs on transgenic parasites. The inhibition of the anti-Pvg37-P2 IgGs on **(A)** male gametocyte exflagellation and **(B)** ookinete formation was assessed by *in vitro* assays. The purified anti-Pvg37-P2 IgGs and pre-immune IgGs were added at concentrations of 0.2, 1.0, and 2.0 μg/μL in culture medium, respectively, incubated with the *TrPvg37Pb* parasites. Data were representative of three separate experiments. The error bar shows mean ± SD. ****P < 0.0001, **P < 0.01, ns, no significance (Student’s t-test). **(C)** The number of oocysts per midgut in mosquitos after 10 days of feeding. N=29, error bar represents mean ± SEM, ** *P < 0.01* (Student’s t-test). **(D)** Mosquito infection rate (oocyst-infected mosquitoes/dissected mosquitoes).

Initially, we investigated the inhibitory activity of anti-Pvg37 IgGs on the formation of exflagellation centers and ookinetes using *in vitro* assays. The inhibitory effects of the anti-Pvg37-P2 IgGs were concentration-dependent. Compared with control IgGs, anti-Pvg37-P2 IgGs at 0.2 μg/μL had no inhibitory effect on exflagellation or ookinete formation (**Figures 4A, B**). However, at 1.0 and 2.0 μg/μL concentrations, anti-Pvg37-P2 IgGs inhibited the number of exflagellation centers by 28.6% and 60.0%, respectively (**Figure 4A**). Similarly, these concentrations of the anti-Pvg37-P2 IgGs reduced the number of ookinetes by 43. 7% and 69.7%, respectively (**Figure 4B**).

The TB potential of anti-Pvg37 IgGs was further assessed through mosquito feeding assays. In a passive antibody transfer experiment, anti-Pvg37-P2 IgGs significantly reduced the number of oocysts per midgut in mosquitoes by 80.2% compared to the control group (**Figure 4C**), although we did not observe noticeable TBA for the anti-Pvg37-P2 IgGs (**Figure 4D**).

### Anti-Pvg37 IgGs exhibit TRA in *P. vivax* clinical isolates

**Figure 5:**
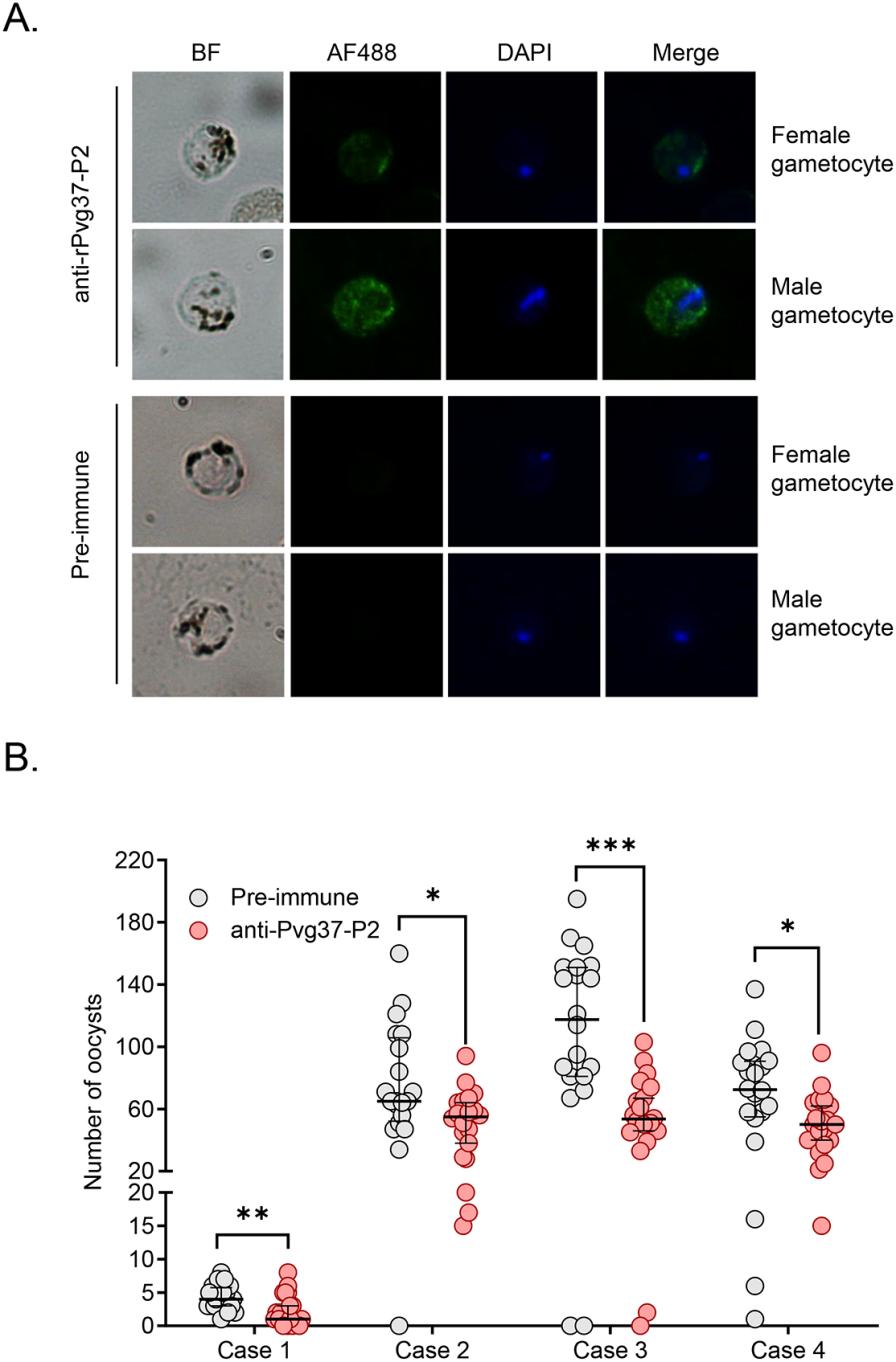
Detection of Pvg37 expression in *P. vivax* gametocytes and TRA of antibody against Pvg37-P2 in DMFA. **(A)** Gametocytes were stained with the purified anti-Pvg37-P2 IgGs and Alexa Fluor 488-conjugated anti-rabbit IgG antibodies. Pre-immune IgGs was used as the negative control. Nuclei were stained with DAPI. BF, bright field; AF488, Alexa Fluor 488; Merge, AF488 + DAPI. **(B)** DMFA was performed using four *P. vivax* isolates with purified IgGs mixed with heat-inactivated (complement minus) AB^+^ human serum in the ratio of 1:1. Numbers of oocysts in mosquito midguts were shown as scatter dot plots. The black horizontal bar indicates the mean number of oocysts in each group. Statistical difference in the mean number of oocysts between the pre-immune and Pvg37 groups was analyzed by the Mann-Whitney U test (*P < 0.05, **P < 0.01, ***P < 0.001).

We studied Pvg37 expression and localization in clinical *P. vivax* samples. In both male and female gametocytes, Pvg37 exhibited cytoplasmic localization and a punctate distribution along the plasma membrane. In contrast, the control IgGs did not show any staining in *P. vivax* gametocytes. These findings demonstrate that anti-Pvg37-P2 IgGs specifically reacted with *P. vivax* gametocytes (**Figure 5A**).

To investigate the efficacy of anti-Pvg37-P2 IgGs in blocking *P. vivax* transmission, DMFA was performed using *P. vivax* samples collected from four Thai patients with *P. vivax* mono-infection, as confirmed by PCR analysis targeting the 18S rRNA using species-specific primers. In DMFA, *An. dirus* mosquitoes feeding on all four blood samples mixed with pre-immune antibodies displayed a mean midgut oocyst intensity of 4.3, 75.4, 111.2, and 69.4, respectively (**Table 1**). In comparison, mosquitoes feeding on the same blood samples mixed with the anti-Pvg37-P2 IgGs at a 2.5 μg/μL concentration showed a mean infection intensity of 2.3, 50.6, 55.2, and 49.6 oocysts per midgut, corresponding to a respective reduction in oocyst density by ~5.9%, 32.9%, 50.4%, and 28.6% (*P* < 0.05, **Figure 5B**; **Table 1**). However, compared to the control antibodies, the anti-Pvg37-P2 IgGs did not show a significant reduction in infection prevalence (**Figure 5B**; **Table 1**).

**Table 1:** Transmission-blocking effect of anti-Pvg37-P2 antibodies for *P. vivax* samples.

*P. vivax* isolatesc	Antibody	Oocyst number median (IQR)	Mean oocysts number ± SEM	% inhibition of oocyst ^a^	P value ^b^	Infection rate (%)	Inf/Diss ^c^	% inhibition of prevalence ^d^	P value ^e^
Case 1	Pre-immune	4.0 (3.0 - 5.8)	4.3 ± 0.4			100	20/20		
	Pvg37-P2	1.0 (1.0 - 4.5)	2.3 ± 0.5	45.9	0.0030	80	16/20	20.00	0.1060
Case 2	Pre-immune	65.0 (52.3 - 105.8)	75.4 ± 8.2			95	19/20		
	Pvg37-P2	55.0 (31.3 - 64.8)	50.6 ± 4.7	32.9	0.0151	100	20/20	–	1.0000
Case 3	Pre-immune	117.5 (81.0 - 151.0)	111.2 ± 11.8			90	18/20		
	Pvg37-P2	53.5 (45.3 - 72.3)	55.2 ± 5.7	50.4	0.0001	95	19/20	–	1.0000
Case 4	Pre-immune	72.5 (55.0 - 90.8)	69.4 ± 7.7			100	20/20		
	Pvg37-P2	50.0 (37.8 - 63.5)	49.6 ± 4.3	28.6	0.0129	100	20/20	–	1.0000

IQR, inter-quartile range; SEM, standard error of mean.

a % inhibition of oocyst intensity was calculated as (mean _control_ – mean _test_)/mean _control_ × 100%.

b for comparing the transmission-blocking effect of anti-Pvg37-P2 IgG with the pre-immune control, the median number of oocyst was statistically analyzed (Mann–Whitney U test) and *P* values less than 0.05 were considered statistically significant.

c The infection prevalence was calculated by number of oocyst-infected mosquitoes per 20 mosquitoes dissected in each group (Inf/Diss).

d % inhibition of prevalence was calculated as % prevalence _contro_- % prevalence _test_.

e Difference between the transmission-blocking effect of anti-Pvg37-P2 and the pre-immune groups was statistically analyzed by Fisher’s exact test. *P* values less than 0.05 were considered statistically significant.

### The Pvg37 sequences are conserved in field *P. vivax* isolates

The genetic diversity of malaria vaccine candidates in endemic parasites poses a challenge to vaccine development (Takala and Plowe, 2009). To investigate whether the variability of TRA among the different isolates might be attributed to genetic polymorphisms of the *pvg37* gene, we sequenced the *pvg37* gene fragments from the four *P. vivax* isolates used in DMFA. Our results showed that these samples had identical amino acid sequences of Pvg37 with the Sal-I strain (**Supplementary Figure S2**).

## Discussion

TBV candidates identified by the MalERA as potential tools for malaria eradication exhibit lower genetic diversity compared to blood or pre-erythrocytic stage antigens, likely due to reduced exposure to human immunity (Alonso et al., 2011; Lopez et al., 2017). However, efforts towards TBV development against *P. vivax*, the second major cause of malaria morbidity, significantly lag behind those targeting *P. falciparum*. All current *P. vivax* TBV candidates (Pvs25, Pvs28, Pvs47, Pvs48/45, Pvs230, and PvHAP2) are orthologs of known *P. falciparum* candidates (Kaslow et al., 1988; Hisaeda et al., 2000; Tachibana et al., 2012, 2015; Qiu et al., 2020). Given the substantial differences in biological characteristics and epidemiology between these two species of *Plasmodium* parasites, methodologies developed for *P. falciparum* TBVs may not always be directly applicable for combating *P. vivax* infection, including the utilization of orthologous vaccine antigens (Mueller et al., 2009). Therefore, it is imperative to identify novel candidate antigens targeted to *P. vivax* to expedite research and development efforts toward an effective TBV.

TBVs elicit antibodies that neutralize the sexual stages of the parasite in blood meals ingested by the *Anopheles* mosquitos, disrupting parasite development in the mosquito and preventing transmission. Upon ingesting the parasite and antibodies, certain antibodies recognize pre-fertilization antigens on the gametocytes/gametes, while others target post-fertilization antigens on zygotes/ookinetes. In malaria-endemic areas, natural antibodies against pre-fertilization antigens exist within populations, providing an immune advantage; however, antibodies targeting post-fertilization antigens may prolong antibody blockade duration (Jones et al., 2015; Dinko et al., 2016; Muthui et al., 2019). Therefore, simultaneous antigens expression during pre- and post-fertilization stages can elicit more significant transmission blockade responses. Our previous study demonstrated that Pbg37 is expressed on the surface of both pre-fertilization (gametes) and post-fertilization (zygotes and ookinetes) stages, suggesting its potential as a candidate antigen for a TBV (Liu et al., 2018). Through functional studies, we determined the importance of Pbg37 during gametocytogenesis, particularly in male gametocyte development. Furthermore, we showed that antiserum against a small 63-amino-acid Pbg37 polypeptide was able to induce moderate TB activity in a mosquito-feeding assay (Liu et al., 2018). Although Pvg37 shares 59% sequence identity with Pbg37 at the amino acid level, it remains unclear if their functional characteristics and expression patterns are consistent across different species.

The biggest challenge facing vaccine development for vivax malaria is the inability to establish long-term *in vitro* cultures of *P. vivax* (da Veiga et al., 2022). However, transgenic rodent malaria parasites expressing a *P. vivax* TBV candidate gene in place of their native genes offer a promising alternative assay system (Ramjanee et al., 2007; Cao et al., 2018). It utilizes the principle that the target cell genome can undergo homologous recombination with the homologous sequences of exogenous DNA to perform precise gene editing or modification, thereby achieving precise manipulation of the target gene (Rocha-Martins et al., 2015). In the current study, we used the transgenic rodent malaria parasites to assess the Pvg37 gene function. We found that Pvg37 was expressed similarly to Pbg37, mainly on the surface of both pre-fertilization (gametes) and post-fertilization (zygotes and ookinetes) stages. The phenotype of *TrPvg37Pb* was similar to the WT line, indicating that *Pvg37* fully restored the defects of *ΔPbg37* parasites during sexual development, demonstrating the potential of this parasite line for evaluating the TB capability of Pvg37.

Recently, peptide-based vaccines have become an attractive alternative approach. These vaccines utilize short protein fragments to induce immune responses against malaria parasites (Skwarczynski et al., 2020). To further elucidate the TB effect of the Pvg37 antibodies, we synthesized two highly conserved and B-cell epitope-rich peptides of Pvg37 to mitigate challenges associated with protein folding, aiming to optimize the functional potential of the protein. Enhanced antibody titers have been shown to correlate with improved TB effects against malaria, particularly in the context of *P. falciparum* (Tachibana et al., 2011, 2012). Studies on antibodies targeting the ookinete surface protein Pfs25 have demonstrated a strong association between high titers and effective TBA, indicating that elevated antibody levels can persist for months while maintaining their blocking efficacy (Kubler-Kielb et al., 2007). In this study, we selected anti-Pvg37-P2 antibodies with higher antibody titers for validation of TRA and TBA. Using the transgenic parasite line, we observed that the anti-Pvg37-P2 IgGs significantly reduced exflagellation and ookinete conversion *in vitro*. Furthermore, an antibody transfer experiment revealed that anti-Pvg37-P2 IgGs led to an 80.2% reduction in oocyst density in mosquitoes. These findings expand upon the TB potential of Pvg37 and highlight the utility of transgenic rodent parasites for evaluating vaccine candidates against *P. vivax*.

The standard membrane feeding assay (SMFA) is currently considered the *in vivo* “gold standard” (Churcher et al., 2012). The DMFA follows a similar design as the SMFA but uses freshly collected gametocyte-infected blood from infected individuals instead of cultured gametocytes to feed and infect mosquitoes (Duffy, 2021). DMFA offers the advantage of testing multiple experimental conditions on a single blood sample, thereby reducing uncontrolled variability. Due to the inability to culture gametocytes for *P. vivax*, DMFA remains the most appropriate method available for this species (Miura et al., 2020). In this study, TRA and TBA for IgGs against Pvg37-P2 were evaluated using DMFA with four clinical *P. vivax* isolates. In DMFA, the transmission reduction rate of anti-Pvg37-P2 IgGs against four clinical *P. vivax* parasites in midgut oocyst density ranged from 28.6% to 50.4%, which is lower than that observed on transgenic strain (80.2%). These findings are consistent with the previous studies (Zhang et al., 2024; Zheng et al., 2024). This disparity may be attributed to various uncontrollable factors of DMFA using field parasite isolates, such as gametocyte density, the proportion of mature gametocytes, the male/female gametocyte ratio, and fertilization pattern among field isolates (Kiattibutr et al., 2017; Ouattara et al., 2024). Certainly, variations in antibody concentrations in the blood meal cannot be overlooked. However, the estimated concentration of purified antibodies in DMFA (~2.5 µg/µL) was higher than that in passively transferred mice (~1.3 µg/µL), suggesting that this difference may not be the primary reason. Additionally, complement might play a role, as passive transfer was performed in mice with complement, while DMFA used purified IgG and inactivated serum. Previous studies show that human complement enhances the TB activity of antibodies against *P. falciparum* and *P. vivax* (Mendis et al., 1987; Quakyi et al., 1987; Healer et al., 1997). Unfortunately, we lacked a positive control for complement in our DMFA and could not directly confirm whether the TB activity of these antibodies depends on complement. The anti-Pvg37 antibody generally elicits a lower TRA compared to existing TBV antigens for *P. vivax* (Qiu et al., 2020; Zheng et al., 2024). However, direct comparison is not appropriate because the proteins used for immunization are expressed using different systems, which affect their immunogenicity and the observed antibody response.

Altogether, both *in vivo* studies with the transgenic parasites in mice and *in vitro* DMFA using clinical *P. vivax* isolates corroborate the TB potential of Pvg37. While our findings suggest that infection prevalence does not significantly decrease, the reduction in oocyst density is crucial. Research has demonstrated that lower oocyst densities hinder the development of P. falciparum in mosquitoes, thereby reducing the number of infectious bites transmitted to humans (Guissou et al., 2023). With fewer oocysts, the likelihood of mosquitoes becoming infective is diminished, ultimately lowering the risk of human infection. Further experimental validation is still required to enhance the TRA and TBA of Pvg37. Peptide-based vaccines often suffer from low immunogenicity, which can be mitigated by developing more advanced adjuvant-based delivery systems. RTS, S/AS02 demonstrated increased antibody titers and augmented cell-mediated immune responses through the utilization of a novel adjuvant (AS02), comprising an oil-in-water formulation containing MPL (a non-toxic derivative of lipopolysaccharide) and QS21 (Garcon et al., 2003). Matrix-M is a promising vaccine adjuvant based on Quillaja saponins, which has demonstrated acceptable safety and the ability to enhance both cellular and humoral immune responses of vaccines (Bengtsson et al., 2013, 2016). Nanoparticle-based platforms, including liposomes, hydrogels, and nanocapsules, can be functionalized for targeted delivery of vaccines (Ouji et al., 2018; Shakeel et al., 2019; Kekani and Witika, 2023; Zhuo et al., 2024). Furthermore, incorporating modified antigens into virus-like particles (VLPs) may augment immunogenicity (Jelínková et al., 2023; Yao et al., 2023). Carrier proteins like Exoprotein A (EPA) have already been successfully employed to elicit enhanced immune responses against TBV candidates (Rausch et al., 2023; Sagara et al., 2023). Additionally, synergistic effects can be achieved by combining multiple stages and antigens in combination vaccines (Sherrard-Smith et al., 2018; Yang et al., 2021). Finally, mRNA vaccines have also exhibited the capacity to induce high levels of antibodies, as evidenced by their impact on Pvs25 (Kunkeaw et al., 2023).

## Data Availability

The datasets presented in this study can be found in online repositories. The names of the repository/repositories and accession number(s) can be found in the article/**Supplementary Material**.
