# Supplementary material for: Evaluation of the transmission-blocking potential of Plasmodium vivax antigen Pvg37 using transgenic rodent parasites and clinical isolates
Source: Front Cell Infect Microbiol. 2025 Jan 24;15:1529770. doi: 10.3389/fcimb.2025.1529770 (PMC11802531; doi:10.3389/fcimb.2025.1529770)

Evaluation of the transmission-blocking potential of *Plasmodium vivax* antigen Pvg37 using transgenic rodent parasites and clinical isolates

Di Zhang ^1†^, Yan Zhao^1†^, Dongyan Liu ^2†^, Fei Liu ^1^, Pengbo Liu ^1^, Biying Zhang ^1^, Zifang Wu ^1^, Wanlapa Roobsoong ^3^, Sirasate Bantuchai ^3^, Sataporn Thongpoon ^3^, Piyarat Sripoorote ^3^, Meilian Wang ^4*^, Liwang Cui ^5*^ and Yaming Cao ^1*^

Note: Supplementary data associated with this article

**Supplementary Table 1.** Polymerase chain reaction (PCR) primer sequences were used in study.

| **Name** | **Sequences (5’ – 3’)** |
| --- | --- |
| TrPvg37Pb identification | |
| Primer 1 | ATATTGAAAAGACCAAATTA |
| Primer 2 | GGGTAAATTTCGCTTCATTGC |
| Primer 3 | CTGGTGCTTTGAGGGGTGAG |
| Primer 4 | GAATGACTCCATCAACAAGA |
| Pvg37 amplification | |
| Pvg37-F | CACTGCGTTTTTCAGATCTGTTTCA |
| Pvg37-R | GTAAAATGGGCACCTAAATGGGATTCG |

**Supplementary Figure 1.** Localization of Pvg37 in *TrPvg37Pb* parasites. Immunofluorescence assays were conducted on *TrPvg37Pb* parasites at various developmental stages using anti-HA mAb. The scale bar represents 5μm.


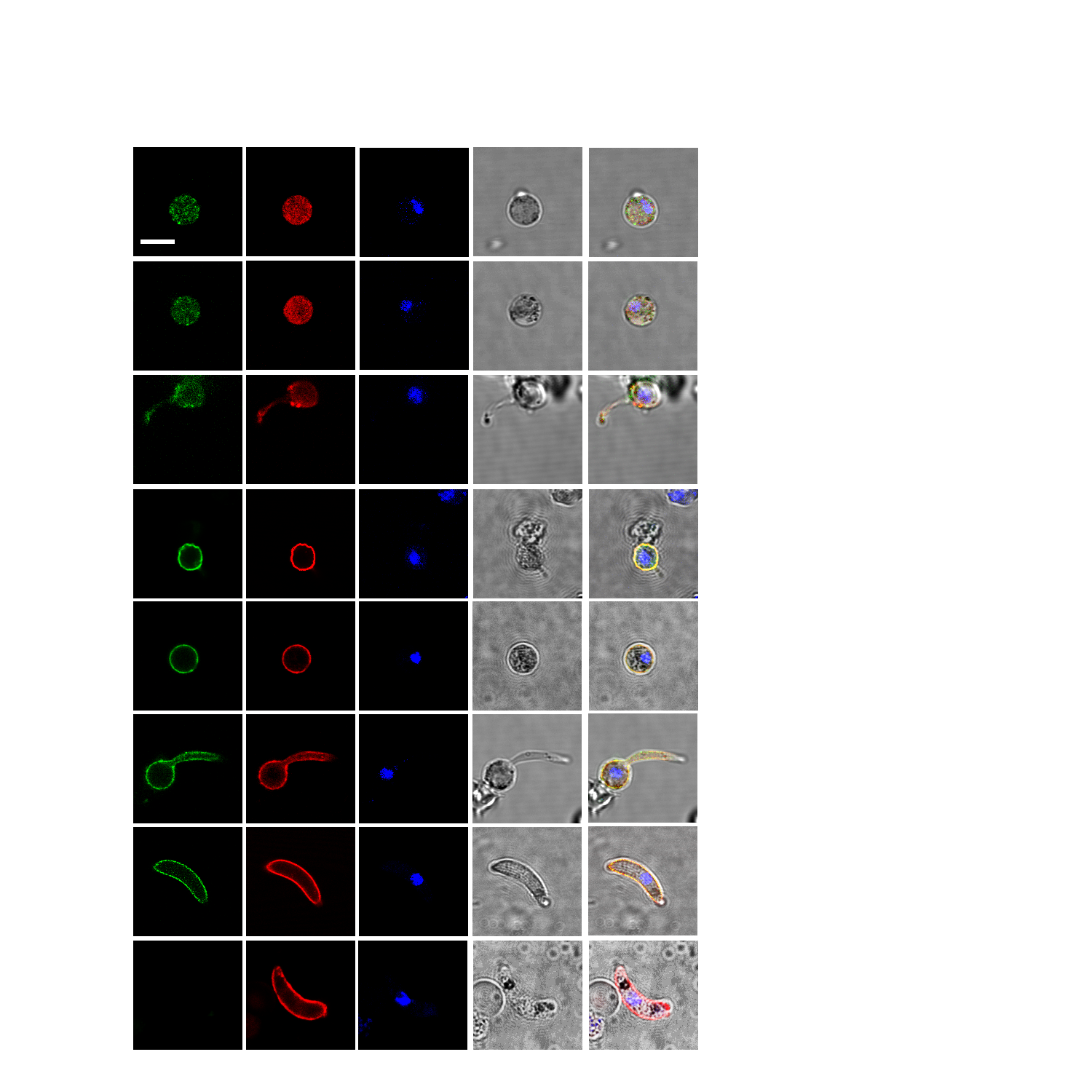


**HA**

**Marker**

**DAPI**

**Merge**

**DIC**

Male

gametocyte

Female gametocyte

Male

gamete

Female gamete

Zygote

Retort

Ookinete

**Pbα-tubulin**

**Pbs47**

**Pbα-tubulin**

**Pbs47**

**Pbs21**

**Pbs21**

**Pbs21**

WT

Ookinete

**Pbs21**

**Supplementary Figure 2.** Multiple sequence alignment was performed for Pvg37 from the *P. vivax* Sal-I strain and four *P. vivax* isolates used in DMFA. The identical amino acid is highlighted in red, while the yellow background indicates the site of non-synonymous mutations. The dotted box indicates the region where Pvg37 peptide 2 (IVHSDESRFTQTQS) is synthesized.


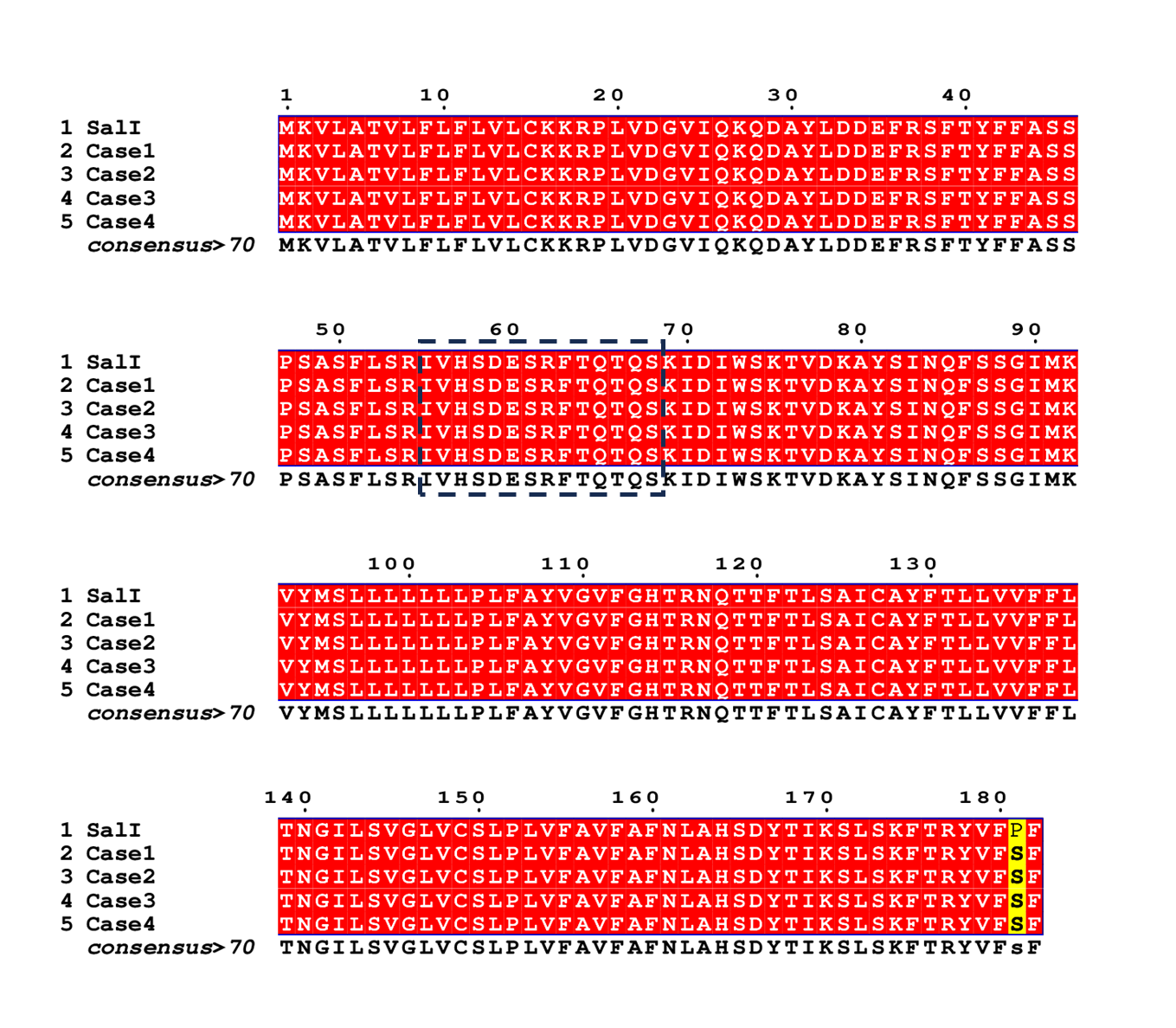

Supplement: Supplementary file 1 [file SupplementaryFile1.docx]
